# Mechanical Performance and Fracture Behavior of Fixed Dental Prostheses Under Bending Loads: An In Vitro Comparative Study

**DOI:** 10.3390/dj14050255

**Published:** 2026-04-28

**Authors:** Cristian Boanca, Dorin Ioan Cocoș, Sergiu Ciprian Focsaneanu, Kamel Earar

**Affiliations:** 1 Faculty of Medicine and Pharmacy, Medical-Pharmaceutical Research Center, “Dunărea de Jos” University of Galati, 800008 Galati, Romania; andiboanca@yahoo.com (C.B.); kamel.earar@ugal.ro (K.E.); 2 Dental-Medicine Department, Faculty of Medicine and Pharmacy, “Dunărea de Jos” University of Galati, 800201 Galati, Romania; 3 Faculty of Material Science and Engineering, University Politehnica of Bucharest, 313 Splaiul Independentei, District 6, 060042 Bucharest, Romania

**Keywords:** fixed dental prosthesis, fracture behavior, fractographic analysis, lithium disilicate, metal-ceramic, micro-computed tomography, three-point bending, zirconia

## Abstract

**Aim:** Fixed dental prostheses (FDPs) are increasingly fabricated from high-strength ceramic materials; however, their fracture behavior under flexurally dominated loading remains incompletely understood. This in vitro study aimed to compare the mechanical performance and fracture mechanisms of four FDP material systems under standardized bending conditions. **Materials and Methods:** Three-unit CAD/CAM-fabricated FDPs were produced from metal-ceramic (P1), zirconia-ceramic (P2), monolithic zirconia (P3), and monolithic lithium disilicate (P4) materials (*n* = 9 per group). Specimens were subjected to three-point bending until failure. Crack initiation load, maximum load, displacement, and stiffness were recorded, and fracture behavior was analyzed using stereomicroscopy, micro-computed tomography (μCT), and scanning electron microscopy (SEM). **Results:** Metal-ceramic FDPs (P1) exhibited the highest crack initiation load (0.89 kN) and maximum load (1.91 kN), with failure predominantly occurring through ceramic veneer delamination without complete framework fracture. Monolithic zirconia FDPs (P3) demonstrated the most brittle failure behavior, characterized by abrupt fracture and unstable crack propagation immediately after crack initiation. Zirconia-ceramic (P2) and lithium disilicate (P4) FDPs showed intermediate mechanical performance, with lithium disilicate exhibiting greater resistance to catastrophic failure (F_max = 0.94 kN) compared with zirconia–ceramic FDPs. **Conclusions:** These findings refine current assumptions regarding the mechanical reliability of monolithic zirconia FDPs under flexural loading and highlight the importance of fracture behavior, rather than peak strength alone, in material selection. Lithium disilicate and metal-ceramic systems exhibited more favorable damage-tolerant responses under static flexural loading. These findings should be interpreted within the limitations of this in vitro model and should not be directly extrapolated to long-term clinical performance.

## 1. Introduction

The growing demand for fixed dental prostheses (FDPs) that combine esthetic quality with mechanical reliability has progressively encouraged a transition from conventional metal-based frameworks toward metal-free restorative systems. Advances in computer-aided design and manufacturing (CAD/CAM) technologies have substantially expanded the range of available chair-side and laboratory-fabricated materials, encompassing high-strength ceramics, high-performance polymers, and fiber-reinforced composite systems [1,2].

Within ceramic restorative systems, yttria-stabilized tetragonal zirconia polycrystal (Y-TZP), commonly referred to as zirconia, has garnered considerable interest due to its favorable mechanical properties, including high flexural strength and enhanced intrinsic fracture toughness resulting from transformation toughening mechanisms [3,4]. Despite these advantages, the elevated elastic modulus and limited optical properties of conventional zirconia have generated concerns related to antagonist wear, biomechanical incompatibility, and esthetic compromise, particularly in anterior applications [5,6]. In response, third-generation zirconia materials with increased cubic phase content have been developed to enhance translucency while preserving sufficient strength for clinical use [7]. However, the concomitant reduction in transformation toughening associated with higher cubic phase fractions has raised questions regarding the long-term mechanical reliability of these newer zirconia formulations [6,8].

Lithium disilicate glass–ceramics constitute a further milestone in the development of metal-free restorative systems. Owing to their distinctive crystalline microstructure, these materials offer a balanced combination of mechanical strength and esthetic performance, promoting crack deflection and effective energy dissipation under functional loading [4]. Consequently, lithium disilicate restorations are commonly indicated for anterior regions and posterior sites subjected to moderate occlusal forces, where predictable performance and enhanced optical properties are required.

Despite the increasing adoption of all-ceramic materials, metal–ceramic prostheses continue to represent a reference standard for long-term mechanical reliability in clinical practice. Their proven performance is primarily related to the synergistic interaction between a ductile metallic framework and a brittle ceramic veneer, which facilitates stress redistribution and reduces the risk of catastrophic failure [9,10,11]. The progressive shift toward metal-free restorative concepts has been enabled by advances in CAD/CAM technologies, allowing for precise, reproducible fabrication and improved structural consistency of prosthetic frameworks [12,13].

Alternative metal-free restorative materials, including high-performance polymers and fiber-reinforced composites, have also been explored in prosthodontics [14,15,16]. However, because these materials were not included in the present experimental design, the current study focused specifically on the comparative mechanical behavior of ceramic-based FDP systems under bending loads.

The present study focuses exclusively on the comparative mechanical evaluation of ceramic-based materials used in three-unit fixed dental prostheses, specifically, conventional metal-ceramic, zirconia-ceramic, monolithic zirconia, and monolithic lithium disilicate systems. Mechanical testing was complemented by pre- and post-fracture micro-computed tomography (μCT) and fractographic analysis under stereomicroscopy, providing a comprehensive assessment of their fracture resistance, mechanical performance, and failure behavior under bending stress. The novelty of this study lies in its mechanism-oriented evaluation of FDP materials under bending loads, integrating crack initiation analysis, stiffness evolution, and multi-scale fracture assessment.

## 2. Materials and Methods

### 2.1. Study Design, Setting, and Ethical Considerations

This study was designed as an in vitro comparative experimental investigation evaluating the mechanical performance and fracture behavior of four types of three-unit fixed dental prostheses (FDPs). The research was conducted between January and May 2024 at the Faculty of Dental Medicine, “Dunărea de Jos” University of Galați, Romania, in collaboration with the Faculty of Dental Medicine, “Grigore T. Popa” University of Medicine and Pharmacy, Iași, Romania.

As the study did not involve human participants or animal subjects, ethical approval was not required, in accordance with institutional and international research guidelines. All laboratory procedures were performed in compliance with institutional safety standards for experimental research on dental materials.

### 2.2. Sample Preparation

Four groups of three-unit fixed dental prostheses (FDPs) were fabricated and tested. Group P1 consisted of conventional metal-ceramic FDPs (Vita VM 13, VITA Zahnfabrik, Bad Säckingen, Germany) veneered on a cobalt–chromium alloy framework (Wirobond C, BEGO, Bremen, Germany). Group P2 consisted of zirconia-ceramic FDPs fabricated using zirconia frameworks (Zirkonzahn Prettau, Zirkonzahn GmbH, Gais, Italy), veneered with feldspathic porcelain (Creation ZI, Creation Willi Geller International GmbH, Meiningen, Austria). Group P3 included monolithic zirconia FDPs (Katana Zirconia HT, Kuraray Noritake Dental Inc., Tokyo, Japan), and Group P4 included monolithic lithium disilicate FDPs (IPS e.max CAD, Ivoclar Vivadent, Schaan, Liechtenstein).

A total of 36 three-unit FDPs were tested (*n* = 36), equally distributed into four groups (*n* = 9 per group): metal-ceramic (P1), zirconia-ceramic (P2), monolithic zirconia (P3), and monolithic lithium disilicate (P4).

All FDPs were constructed following standard digital design and CAD/CAM milling protocols. All specimens were generated from the same master digital design to standardize geometry across material groups. The prostheses reproduced a posterior three-unit FDP configuration with identical abutment dimensions and pontic morphology. The mesial and distal connectors were standardized to a minimum cross-sectional area of 9.0 mm^2^, measured at the narrowest portion of the connector, with a height of 4.0 mm and a width of 2.25 mm. The pontic dimensions were standardized as follows: mesiodistal length 10.0 mm, buccolingual width 8.0 mm, and occlusogingival height 7.0 mm. In the bilayer restorations, framework thickness was maintained at 0.8 mm, while veneering ceramic thickness was limited to 1.0 mm in the veneered anatomical regions. These dimensions were kept constant across all groups to minimize geometry-related variability during mechanical comparison. Ceramic veneering (where applicable) was performed under manufacturer-recommended firing cycles. Specimens were stored in distilled water at 37 °C for 24 h before testing.

### 2.3. Mechanical Testing

All specimens were subjected to three-point bending using a universal testing machine (Instron 34TM-10, Instron Corp., Norwood, MA, USA). Tests were performed under displacement-controlled conditions at a crosshead speed of 1 mm/min and continued until catastrophic failure. The span length between the supporting rollers was standardized at 20 mm for all specimens. No abutment simulation or cementation protocol was used in the present study. Each FDP was tested as a free-standing structure directly supported on the metallic rollers of the three-point bending device. The support points were positioned symmetrically beneath the terminal retainer regions, while the loading piston was centered over the pontic to ensure consistent load application and reproducible boundary conditions. This setup was selected as a standardized structural bending model for comparative mechanical testing rather than a full simulation of the clinical abutment-restoration complex. Mechanical testing was conducted at room temperature under dry conditions.

The following mechanical parameters were extracted from the force–displacement curves:

- F_1_ (kN): Load at initial crack formation;
- DF_1_ (mm): Displacement at initial crack formation;
- F_max (kN): Maximum load before failure;
- DF_max (mm): Displacement at maximum load;
- R (kN/mm): Stiffness, calculated from the slope of the initial linear elastic portion of the force–displacement curve before the first detectable deviation associated with crack initiation.

### 2.4. Micro-CT Imaging

Each specimen was scanned using a micro-computed tomography (μCT) system (XT H–225 Helical CT Scanner, Amstelveen, The Netherlands), both before and after mechanical loading. Scanning was performed with a voxel resolution of 10–15 μm, allowing the detection of pre-existing internal defects, crack initiation sites, crack propagation paths after failure, and volumetric changes. Image acquisition was carried out using standard scanning parameters recommended by the manufacturer for high-resolution analysis of dental ceramic materials. Volume reconstruction and qualitative analysis were performed using dedicated software to correlate internal damage patterns with mechanical testing results.

### 2.5. Fractographic Analysis

Post-fracture analysis was performed to evaluate the fracture patterns and failure mechanisms of the tested fixed dental prostheses. Fractured specimens were examined using a stereomicroscope (SMZ 1270, Nikon, Amstelveen, The Netherlands). The analysis focused on identifying the fracture origin, crack initiation sites, crack propagation direction, and overall fracture surface morphology.

Special attention was given to material-specific fractographic features, including the presence of mirror, mist, and hackle regions, as well as veneer delamination patterns in bilayered prostheses. Fracture initiation was evaluated in relation to the tensile zone generated during three-point bending, particularly at areas of geometric transition and support–pontic interfaces. Fractographic observations were qualitatively correlated with mechanical test results and micro-computed tomography findings to provide a comprehensive interpretation of material-dependent fracture behavior and stress distribution under bending loads.

### 2.6. Scanning Electron Microscopy (SEM) Analysis

Fractured specimens were cleaned ultrasonically in ethanol for 5 min, air-dried, and then sputter-coated with a thin gold layer (~10 nm) to ensure surface conductivity during SEM observation. Fractured specimens from each group were analyzed using a scanning electron microscope (Quattro S SEM, ThermoFisher Scientific, Waltham, MA, USA). SEM imaging was conducted to examine surface morphology at high resolution and to identify microstructural features such as crack origin points, intergranular fracture paths, and evidence of adhesive or cohesive failure modes. Images were captured at magnifications ranging from ×100 to ×2000, depending on the region of interest and the level of detail required for fracture surface analysis.

### 2.7. Statistical Analysis

Descriptive statistical analysis was performed for all mechanical parameters, including crack initiation load (F_1_), maximum load (F_max), displacement at crack initiation and at maximum load, and stiffness (R). Data are presented as mean ± standard deviation, providing an overview of central tendency and variability within each experimental group.

Normality of data distribution was assessed using the Shapiro–Wilk test. Depending on data distribution, intergroup comparisons were conducted using one-way ANOVA or the Kruskal–Wallis test, with Tukey’s or Dunn’s post hoc tests applied as appropriate. Omnibus intergroup comparisons were performed using one-way ANOVA when data met normality assumptions. To complement significance testing, between-group effects were quantified primarily as mean differences with 95% confidence intervals (CI). Standardized effect sizes (Hedges’ g) were computed but interpreted cautiously because several comparisons showed near-complete distributional separation driven by large between-group differences relative to low within-group variability, which can yield extremely large standardized estimates.

All data processing, statistical analyses, and graphical representations were performed using GraphPad Prism software (version 10.0; GraphPad Software, San Diego, CA, USA).

A priori sample size estimation was performed based on effect magnitudes reported in previous in vitro fracture resistance studies of fixed dental prostheses under flexural loading. Considering a large expected effect size (f ≥ 0.40), a significance level of α = 0.05, and a desired statistical power of 80%, the minimum required sample size was calculated as eight specimens per group for one-way ANOVA comparisons. Therefore, nine specimens per group were included to ensure adequate statistical sensitivity and compensate for potential variability.

## 3. Results

The results of the present study are organized to reflect the sequential evaluation of the mechanical performance and fracture behavior of the tested fixed dental prostheses. Mechanical testing outcomes are presented first, followed by micro-computed tomography findings and fractographic observations. This integrated approach allows a direct correlation between load–displacement behavior, internal damage patterns, and material-specific fracture mechanisms.

### 3.1. Mechanical Tests

As shown in Table 1, mechanical parameters are reported as mean ± standard deviation (SD) for each experimental group (*n* = 9 per group). F_1_ denotes the load at initial crack formation, DF_1_ the displacement at crack initiation, F_max the maximum load before catastrophic failure, DF_max the displacement at maximum load, and R the stiffness, calculated as the slope of the initial linear portion of the force–displacement curve before crack initiation during three-point bending. All specimens were tested under identical experimental conditions, using a standardized span length and loading rate. Omnibus statistical testing revealed significant material-dependent differences for the evaluated mechanical parameters (*p* < 0.05).

Stiffness values also differed significantly among the tested groups (Table 1). P1 exhibited the highest mean stiffness (5.01 ± 1.12 kN/mm), followed by P4 (3.87 ± 1.08 kN/mm), P3 (3.21 ± 1.44 kN/mm), and P2 (2.98 ± 0.96 kN/mm). These findings indicate that the metal-ceramic FDPs provided the greatest resistance to deformation under initial linear loading, whereas zirconia-ceramic specimens showed the lowest stiffness. The higher stiffness observed in P1 is consistent with the presence of a metallic framework, while the intermediate values recorded for P3 and P4 suggest material-dependent differences in structural response under static flexural loading.

**Scheme 1:**
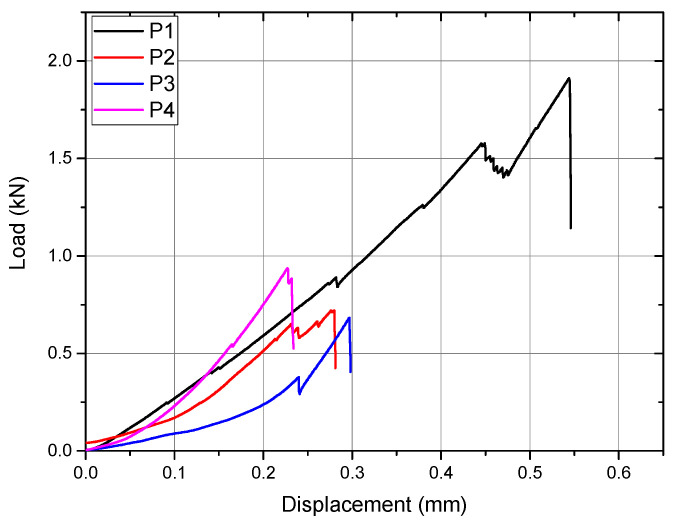
Representative force–displacement curves obtained during three-point bending tests for the four FDP groups (P1–P4), illustrating the typical mechanical response of each material system.

Representative force–displacement curves obtained during three-point bending tests are shown in Scheme 1 for the four FDP material systems (P1–P4). The curves illustrate material-dependent mechanical response under standardized flexural loading, including differences in crack initiation, load-bearing capacity, and post-initiation failure behavior. P1 showed the highest crack initiation force and overall load-bearing capacity, whereas P3 exhibited a more abrupt brittle fracture pattern. P2 and P4 displayed intermediate mechanical behavior, with P4 showing greater resistance to catastrophic failure in this experimental model than P2.

Table 2 presents pairwise comparisons between the FDP material groups for the main mechanical parameters. Δ represents the mean difference between the first-listed and second-listed groups. The percentage difference (%Δ) was calculated relative to the second-listed group. Mean ratio represents the ratio of the first-listed to the second-listed group means (e.g., P1/P3). Confidence intervals (95% CI) are reported for the mean difference. P-values were derived from Tukey’s multiple comparison test following one-way ANOVA. One-way ANOVA demonstrated significant overall material-dependent effects for F_1_ (η^2^ = 0.967), F_max (η^2^ = 0.983), DF_max (η^2^ = 0.949), and stiffness R (η^2^ = 0.341), indicating extremely large effects for crack initiation load, maximum load, and displacement at failure, and a moderate-to-large effect for stiffness.

Initial crack resistance (F_1_) was highest in group P1, reflecting superior resistance to crack initiation under bending loads. Groups P2 and P4 exhibited comparable crack initiation behavior, while P3 demonstrated the lowest resistance to initial crack formation. A similar material-dependent hierarchy was observed for maximum failure load (F_max), with P1 showing the highest load-bearing capacity, followed by P4, P2, and P3. Pairwise post hoc comparisons confirmed significant differences for most contrasts involving P1, whereas some comparisons between intermediate-performing groups were smaller or not statistically significant.

### 3.2. Micro-CT Results

**Figure 1:**
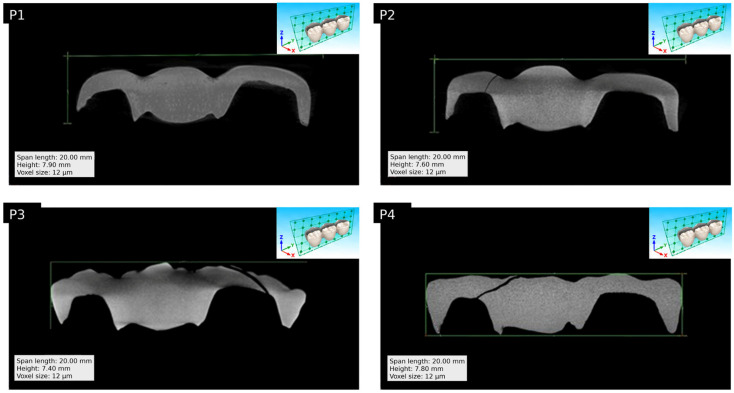
(**P1**–**P4**) Post-fracture micro-CT reconstructions of the four FDP groups after mechanical testing.

Post-fracture micro-CT reconstructions of representative FDP specimens from the four experimental groups are presented in Figure 1 (P1–P4). The images illustrate material-dependent internal fracture patterns after three-point bending. P1 shows veneer delamination without complete framework failure, whereas P2 and P4 exhibit more extended crack propagation involving the support–pontic region. P3 demonstrates a predominantly brittle fracture pattern with limited crack branching. All specimens were scanned using identical parameters (voxel size: 12 μm).

### 3.3. Fractographic Observations

**Figure 2:**
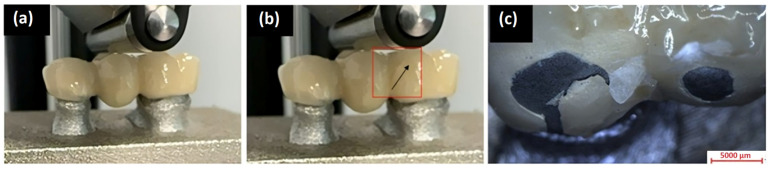
(**a**–**c**) Fracture progression in a metal-ceramic FDP during mechanical testing. The red frame highlights the region of crack initiation, while the arrow indicates the direction/site of crack propagation in the tensile zone.

Fractographic analysis indicated that fracture initiation in all specimens occurred under bending-induced tensile stress, with material-dependent failure patterns. In group P1, a more uniform stress distribution was observed (Figure 2a–c), attributable to the presence of the metallic substructure. Crack initiation consistently occurred near curvature transition zones at the pontic junctions and propagated primarily within the ceramic veneer. Failure was dominated by veneer delamination rather than by bulk framework fracture, reflecting effective stress redistribution and partial energy dissipation before catastrophic failure. As shown in Figure 2, panel (a) illustrates the test setup under standardized loading conditions, panel (b) shows crack initiation at 0.89 kN near the middle–right pontic junction, and panel (c) presents the post-fracture detail with veneer delamination and fracture origin in the tensile zone, consistent with tension-induced failure without complete framework fracture.

**Figure 3:**
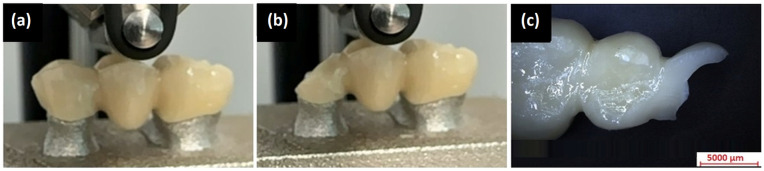
(**a**–**c**) Fractographic sequence showing failure progression in a tested FDP specimen.

**Figure 4:**
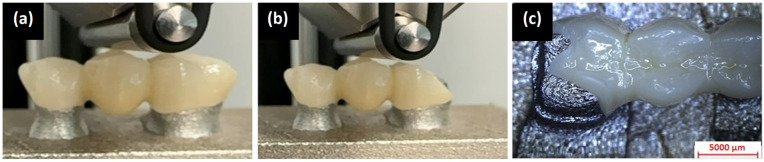
(**a**–**c**) Failure sequence of a P3 sample illustrating stress distribution and fracture path.

**Figure 5:**
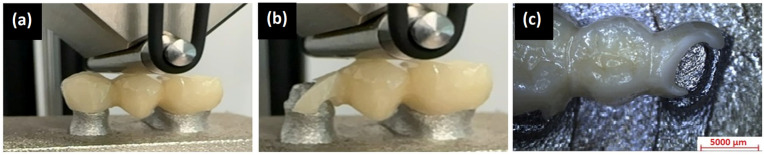
(**a**–**c**) Fracture sequence in lithium disilicate sample (P4).

Groups P2, P3, and P4 exhibited fracture initiation at the support–prosthesis contact region, as illustrated in Figure 3, Figure 4 and Figure 5. In P2 (Figure 3a–c), crack initiation was predominantly observed in the left pontic area, corresponding to the tensile zone generated during three-point bending. As shown in Figure 3, panel (a) illustrates the test setup under standardized loading conditions, panel (b) shows crack initiation in the left pontic region, corresponding to the tensile zone generated during bending, and panel (c) presents the post-fracture view with the resulting crack propagation pattern in the pontic area.

In P3 (Figure 4a–c), crack initiation was more frequently observed in the right pontic region, corresponding to the tensile stress concentration zone under three-point bending. This asymmetric initiation pattern may reflect local geometric factors or internal structural heterogeneities. As shown in Figure 4, panel (a) presents the specimen under standardized loading conditions before visible fracture, panel (b) shows crack initiation in the right pontic region, corresponding to the tensile stress concentration zone under bending, and panel (c) illustrates the post-fracture view with crack propagation from the support–bridge interface toward the outer prosthetic contour.

A comparable fracture initiation pattern was observed in P4 (Figure 5a–c), with crack origin localized in the tensile region adjacent to the support interface under three-point bending. As shown in Figure 5, panel (a) illustrates the test setup under standardized loading conditions, panel (b) shows crack initiation adjacent to the support interface in the tensile region, and panel (c) presents the post-fracture view with the resulting crack propagation pattern through the prosthetic structure.

Across all ceramic groups, fractures propagated along multiple planes, and whitening zones adjacent to the main crack path indicated micro-crack formation before complete failure. The overall mode of fracture was abrupt and tensile in nature, typical of brittle materials lacking a ductile substructure.

### 3.4. SEM Analysis

**Figure 6:**
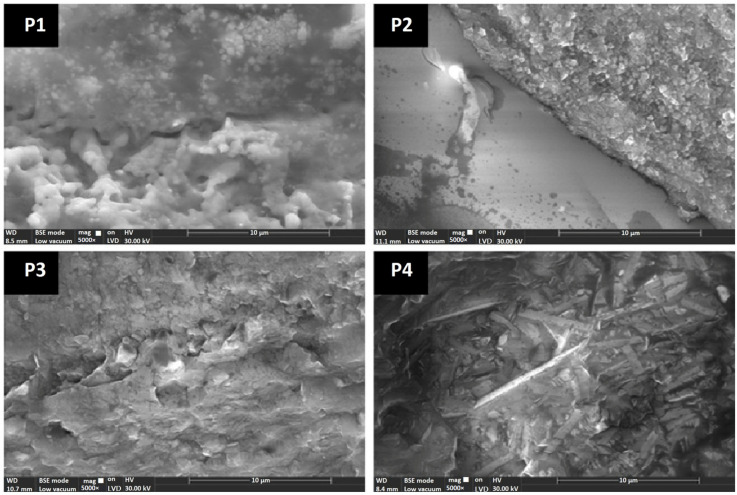
SEM fractographic images of FDP specimens (**P1**–**P4**).

SEM analysis (Figure 6, P1–P4) provided a high-resolution visualization of fracture surface morphology. In P1, veneer delamination at the metal-ceramic interface was evident. P2 exhibited mixed adhesive-cohesive fracture patterns with secondary crack formation. P3 demonstrated intergranular brittle fracture with limited crack branching, whereas P4 showed rough fracture surfaces with microcrack networks, consistent with enhanced energy dissipation before final failure.

In P1, layered failure characterized by veneer delamination and step-like interfacial features was observed, accompanied by hackle lines indicating crack initiation on the tensile side. P3 demonstrated smooth mirror regions surrounded by mist and hackle zones, consistent with predominantly intergranular brittle fractures. P2 exhibited mixed adhesive–cohesive failure with irregular fracture planes, whereas P4 showed rougher fracture surfaces with deflected cracks and microcrack networks, suggestive of greater energy dissipation before catastrophic failure. These SEM observations corroborate the mechanical and μCT findings, confirming material-dependent differences in fracture behavior and failure mode among the tested groups.

Based on the mechanical and fractographic findings, P1 demonstrated the highest performance in terms of crack initiation load and maximum load, maintaining structural integrity over a greater displacement range. P4 and P2 exhibited intermediate mechanical behavior, with P4 showing slightly greater resistance to catastrophic failure. P3 demonstrated the most brittle fracture response, characterized by limited damage tolerance and unstable crack propagation following crack initiation. Overall, under static flexural loading conditions, P1 showed the most favorable mechanical response among the tested systems, whereas P3 exhibited the lowest resistance to crack initiation and ultimate failure within the experimental framework of this study.

## 4. Discussion

### 4.1. Influence of Material Composition on Mechanical Performance

The mechanical behavior of FDPs under bending loads is influenced by their composition, structure, and modulus of elasticity. The superior performance of P1 (metal-ceramic) is attributed to the combination of a ductile metallic framework and a brittle ceramic veneer, which promotes stress redistribution and delays veneer failure. This is consistent with clinical findings showing that metal-ceramic FDPs have high long-term survival rates [3,7].

In contrast, P3 exhibited limited damage tolerance and brittle fracture behavior. Although zirconia has high compressive strength, it lacks the energy absorption capacity of ductile materials, and once a crack initiates, it propagates rapidly [8]. This aligns with the observed sudden failure after first crack initiation, with minimal increase in displacement.

P2 and P4 bridges behaved more similarly, with more gradual failure and improved crack resistance compared with P3. Notably, P4 showed slightly better performance in terms of maximum load before failure, which may reflect the ability of its glass-ceramic network to dissipate energy before crack propagation [9].

The stiffness data further support the overall mechanical findings. P1 showed the highest mean stiffness, indicating greater resistance to deformation during initial loading, which is consistent with the reinforcing effect of the metallic framework. P2 exhibited the lowest stiffness, whereas P3 and P4 showed intermediate values. These results suggest that structural rigidity under bending is strongly influenced by material composition and prosthetic architecture. However, stiffness should be interpreted together with crack initiation load, maximum load, and fracture pattern, since a higher stiffness value does not necessarily correspond to greater damage tolerance after crack initiation.

Fractographic observations confirm that stress concentration at support interfaces is a common initiation site, especially for ceramic-only restorations. The metal-ceramic sample displayed a more predictable failure pattern with veneer delamination due to tensile stresses in the ceramic rather than total bulk fracture.

The present findings are consistent with previous studies showing material-dependent differences in the mechanical behavior of FDPs. The superior fracture resistance of metal-ceramic FDPs (P1) agrees with the observations of Preis et al. [1], who reported that metal substructures redistribute tensile stresses and delay catastrophic veneer failure, as well as with more recent review evidence supporting the long-term mechanical reliability of metal-ceramic systems compared with fully ceramic alternatives [3].

The brittle failure pattern observed in monolithic zirconia (P3) is consistent with recent literature describing monolithic and translucent zirconia as materials with high stiffness, limited deformation capacity, and greater susceptibility to abrupt crack propagation under tensile- or flexural-dominated loading [4,7,8]. This interpretation is further supported by the reduced transformation toughening reported for newer translucent zirconia formulations [7,8].

The intermediate mechanical performance of lithium disilicate FDPs (P4) agrees with the results of Karevan et al. [15], who highlighted the favorable energy dissipation and more gradual crack propagation typical of glass-ceramic systems. Overall, the current results indicate that metal-ceramic FDPs showed the highest mechanical stability in the present model, whereas lithium disilicate provided a more balanced combination of strength and damage tolerance.

### 4.2. Fracture Mechanisms and Stress Distribution Under Bending Loads

These results suggest that, within the limits of this static in vitro flexural model, metal-ceramic and lithium disilicate restorations exhibited more favorable damage-tolerant mechanical behavior than monolithic zirconia. While full-contour zirconia performs reliably under compressive stress, it appears more susceptible to tensile-dominated or bending-induced failure modes typical of fixed dental prostheses. In line with current trends in the medical and dental literature emphasizing a broader biological and functional framework for restorative material evaluation, mechanical findings should be interpreted alongside interdisciplinary evidence rather than in isolation [17,18,19].

This analysis highlights the role of material microstructure, elastic mismatch, and crack propagation behavior in determining the mechanical response of fixed dental prostheses under three-point bending. Metal-ceramic restorations benefit from the presence of a ductile metallic substructure, which promotes stress redistribution and progressive crack stabilization before catastrophic failure, thereby limiting abrupt crack propagation and increasing damage tolerance within the ceramic veneer layer [20,21,22]. This behavior is consistent with fracture mechanics principles describing the ability of compliant substructures to reduce stress intensity at the crack tip.

In contrast, monolithic zirconia demonstrates a predominantly brittle fracture response under tensile and flexural loading, owing to its high elastic modulus and limited capacity for plastic deformation. Once crack initiation occurs, rapid crack propagation may ensue when the applied stress intensity factor exceeds the intrinsic fracture toughness (K_IC) of the material [23,24]. Although fracture toughness was not directly measured in the present study, the observed abrupt post-initiation behavior is consistent with brittle crack instability described in polycrystalline ceramics [23,24].

Lithium disilicate and zirconia-reinforced glass-ceramic systems demonstrate intermediate fracture behavior, where crack deflection, microcracking, and limited transformation-related mechanisms contribute to a more gradual failure pattern and enhanced resistance to crack growth when compared with monolithic zirconia [25,26,27,28]. The presence of interlocking lithium disilicate crystal phases within a glassy matrix creates effective microstructural barriers to crack propagation, resulting in more distributed stress fields and greater structural crack stability before ultimate fracture [20,26].

From a mechanistic perspective, stress distribution under bending loads is strongly influenced by elastic mismatch in bilayer systems [21,22]. In these structures, tensile stresses tend to concentrate at ceramic surfaces and material interfaces, whereas compressive regions may partially retard crack growth [21,22]. These interactions emphasize that fracture behavior cannot be adequately characterized by peak load values alone. Instead, parameters related to crack stability and post-initiation structural response provide more reliable insight into failure mechanisms, particularly in the presence of processing-induced defects and residual stresses [22,23,24].

Accordingly, while metal-ceramic and lithium disilicate-based restorations tend to exhibit more damage-tolerant and progressive fracture patterns under flexural loading, monolithic zirconia remains more susceptible to unstable brittle fracture with limited warning, especially under tensile-dominated loading conditions where transformation toughening or crack-bridging effects are minimal [23,24,27,29]. These mechanistic considerations highlight the importance of interpreting in vitro bending tests within a fracture mechanics framework. This is particularly important when relating material behavior to functional performance.

### 4.3. Study Strengths, Limitations, and Future Perspectives

The main strength of the present study lies in its standardized and rigorously controlled methodological design, which enabled a reproducible comparison of different fixed dental prosthesis (FDP) material systems under identical loading conditions. The use of CAD/CAM-fabricated three-unit FDPs ensured geometric consistency, while the combination of three-point bending tests, micro-computed tomography (μCT), and scanning electron microscopy (SEM) allowed a multi-scale evaluation of fracture behavior. This integrated approach facilitated correlation between mechanical response, internal damage evolution, and fracture surface morphology, providing insight into crack initiation, propagation patterns, and energy dissipation mechanisms.

Although cyclic fatigue and thermomechanical aging protocols are commonly employed to approximate intraoral conditions, the present investigation intentionally focused on static three-point bending as a controlled mechanistic baseline model. Static flexural loading enables precise identification of crack initiation thresholds, stiffness evolution, and failure modes under tensile-dominated stress while minimizing confounding variables associated with cumulative damage and environmental degradation. By isolating material-dependent structural responses under standardized boundary conditions, this approach allows direct comparison of fracture behavior among different FDP systems.

The primary objective of this study was not to predict long-term clinical survival but to elucidate comparative fracture mechanisms and damage tolerance under flexural loading. While cyclic fatigue and thermomechanical aging are essential for clinical extrapolation, understanding crack initiation and unstable propagation represents a fundamental prerequisite for interpreting fatigue-driven failure. Future investigations incorporating dynamic loading and environmental aging protocols will be necessary to extend these mechanistic findings toward clinical performance prediction.

Nevertheless, several limitations must be acknowledged. The in vitro design does not fully replicate intraoral conditions, such as cyclic loading, thermal fluctuations, humidity, or chemically active environments, all of which may influence long-term restoration performance. The absence of fatigue and aging protocols may therefore underestimate the contribution of slow crack growth and cumulative damage in ceramic-based systems. Accordingly, the present findings should be interpreted as descriptive and comparative rather than predictive of long-term clinical outcomes.

A further limitation is that fracture toughness (K_IC) was not directly measured using standardized methods (e.g., ASTM or ISO-based fracture toughness testing). Instead, the study adopted a structure-level, mechanism-oriented approach, combining crack initiation load, stiffness evolution, and multi-scale fractographic analysis (stereomicroscopy, μCT, SEM) to characterize system-level fracture behavior and damage tolerance of three-unit FDPs under flexural loading. Therefore, fracture-related interpretations herein refer to structural crack stability and post-initiation load-bearing capacity rather than intrinsic material fracture toughness (K_IC). Future research should incorporate standardized fracture toughness measurements together with fatigue testing to further bridge structural behavior and intrinsic material properties.

Therefore, the present findings should be interpreted as comparative mechanistic evidence obtained under static in vitro flexural loading, rather than as direct predictors of long-term clinical performance.

Future investigations should include larger sample sizes to enhance external validity and integrate fatigue testing, thermomechanical aging, and environmental conditioning to improve clinical relevance. Expanding the experimental framework to include high-performance polymer-based and hybrid materials (e.g., PEEK, PEKK, fiber-reinforced composites) may further clarify alternative strategies for stress modulation in FDPs. The integration of finite element analysis and fracture mechanics modeling could additionally improve understanding of stress distribution and failure mechanisms, supporting optimized material selection and prosthetic design.

## 5. Conclusions

This study provides a mechanism-based comparison of FDP materials under static bending loads, extending beyond maximum load values to include crack initiation, stiffness evolution, and failure mode. P1 demonstrated the highest fracture resistance and post-crack load-bearing capacity under static flexural loading, indicating a more favorable structural response within the present experimental model. In contrast, P3, despite its high strength, exhibited limited damage tolerance and abrupt brittle fracture following crack initiation. P2 and P4 showed intermediate mechanical performance, with P4 displaying greater resistance to catastrophic failure. Overall, these findings emphasize that material-related differences in FDP performance should be interpreted not only in terms of peak strength, but also in relation to fracture behavior and structural response under standardized static flexural loading. Direct clinical extrapolation should be made with caution.

## Figures and Tables

**Table 1:** Mechanical test results for the four FDP groups under three-point bending.

Group	F_1_ [kN]	DF_1_ [mm]	F_max [kN]	DF_max [mm]	Stiffness R [kN/mm]
P1	0.869 ± 0.029	0.282 ± 0.021	1.912 ± 0.084	0.544 ± 0.037	5.01 ± 1.12
P2	0.571 ± 0.034	0.215 ± 0.018	0.720 ± 0.062	0.280 ± 0.026	2.98 ± 0.96
P3	0.378 ± 0.041	0.240 ± 0.020	0.683 ± 0.058	0.297 ± 0.031	3.21 ± 1.44
P4	0.547 ± 0.033	0.165 ± 0.016	0.938 ± 0.071	0.227 ± 0.024	3.87 ± 1.08
*p*-value (one-way ANOVA)	<0.001	<0.001	<0.001	<0.001	0.004

Abbreviations: F_1_, load at initial crack formation; DF_1_, displacement at crack initiation; F_max, maximum load before failure; DF_max, displacement at maximum load; R, stiffness.

**Table 2:** Pairwise comparisons between FDP material groups for key mechanical parameters under three-point bending.

Mechanical Parameter	Comparison	Δ (Mean Difference)	%Δ	Mean Ratio	95% CI	*p*-Value
F_1_ (kN)	P1 vs. P2	0.298	+52%	1.52	0.254–0.342	<0.001
	P1 vs. P3	0.491	+130%	2.30	0.455–0.527	<0.001
	P1 vs. P4	0.322	+59%	1.59	0.291–0.353	<0.001
	P2 vs. P3	0.193	+51%	1.51	0.149–0.237	<0.001
	P2 vs. P4	0.024	+4%	1.04	−0.020–0.068	0.464
	P3 vs. P4	−0.169	−31%	0.69	−0.213 to −0.125	<0.001
F_max (kN)	P1 vs. P2	1.192	+166%	2.66	1.114–1.270	<0.001
	P1 vs. P3	1.229	+180%	2.80	1.156–1.302	<0.001
	P1 vs. P4	0.974	+104%	2.04	0.896–1.052	<0.001
	P2 vs. P3	0.037	+5%	1.05	−0.041–0.115	0.590
	P2 vs. P4	−0.218	−23%	0.77	−0.296 to −0.140	<0.001
	P3 vs. P4	−0.255	−27%	0.73	−0.333 to −0.177	<0.001
DF_max (mm)	P1 vs. P2	0.264	+94%	1.94	0.232–0.296	<0.001
	P1 vs. P3	0.247	+83%	1.83	0.215–0.279	<0.001
	P1 vs. P4	0.317	+140%	2.40	0.285–0.349	<0.001
	P2 vs. P3	−0.017	−6%	0.94	−0.049–0.015	0.503
	P2 vs. P4	0.053	+23%	1.23	0.021–0.085	0.006
	P3 vs. P4	0.070	+31%	1.31	0.038–0.102	<0.001
Stiffness R (kN/mm)	P1 vs. P2	2.03	+68%	1.68	0.930–3.130	0.002
	P1 vs. P3	1.80	+56%	1.56	0.700–2.900	0.009
	P1 vs. P4	1.140	+29%	1.29	0.040–2.240	0.031
	P2 vs. P3	−0.23	−7%	0.93	−1.330–0.870	0.945
	P2 vs. P4	−0.89	−23%	0.77	−1.990–0.210	0.168
	P3 vs. P4	−0.66	−17%	0.83	−1.860–0.540	0.457

## Data Availability

The original contributions presented in this study are included in the article. Further inquiries can be directed to the corresponding authors.

## Abbreviations

The following abbreviations are used in this manuscript:

Abbreviation	Definition
ASTM	American Society for Testing and Materials
CAD	Computer-Aided Design
CAD/CAM	Computer-Aided Design/Computer-Aided Manufacturing
CAM	Computer-Aided Manufacturing
Co-Cr	Cobalt-Chromium
DF_max	Displacement at Maximum Load
DF_1_	Displacement at Initial Crack Formation
E.max	Monolithic Lithium Disilicate
FDP	Fixed Dental Prosthesis
FDPs	Fixed Dental Prostheses
FRC	Fiber-Reinforced Composite
F_max	Maximum Load Before Failure
Full-Zr	Monolithic Zirconia
F_1_	Load at Initial Crack Formation
HT	High Translucency
ISO	International Organization for Standardization
MC	Metal-Ceramic
PAEK	Polyaryletherketone
PEEK	Polyetheretherketone
PEKK	Polyetherketoneketone
R	Stiffness
SEM	Scanning Electron Microscopy
Y-TZP	Yttria-stabilized Tetragonal Zirconia Polycrystal
Y_2_O_3_	Yttrium Oxide
ZrC	Zirconia-Ceramic
μCT	Micro-Computed Tomography
